# The association between sports participation and fertility intentions among Chinese youth: an empirical study based on the China family panel studies

**DOI:** 10.3389/fpubh.2026.1904111

**Published:** 2026-10-02

**Authors:** Wei Zhao, Guangwu Dong, Dongsheng Liu, Chunhui Yan

**Affiliations:** 1 Sports Engineering Faculty, Belarusian National University of Technology, Minsk, Belarus; 2 College of Sports Science, Nanjing Normal University, Nanjing, China; 3 College of Physical Education, Shihezi University, Shihezi, Xinjiang, China

**Keywords:** China Family Panel Studies (CFPS), fertility intention, Confidence in the future, life satisfaction, sports participation

## Abstract

**Objective:**

This study aimed to examine the association between sports participation and fertility intentions among Chinese youth and to assess the potential mediating roles of life satisfaction and confidence in the future.

**Methods:**

Data were drawn from the 2018 and 2022 waves of the China Family Panel Studies (CFPS). A serial mediation model was specified to examine the potential psychological pathways linking sports participation to fertility intentions. Two-way fixed-effects regression models were used to assess the statistical association between sports participation and fertility intentions. The significance of the specific indirect effects and the serial indirect effect was evaluated using an individual-level cluster bootstrap procedure with 5,000 resamples.

**Results:**

Sports participation was significantly and positively associated with fertility intentions (*β* = 0.046, *p* < 0.01). Mediation analysis revealed that the total indirect effect of life satisfaction and future confidence was 0.015 (95% CI = [0.009, 0.020]). Specifically, the independent indirect effect of life satisfaction was 0.007 (95% CI = [0.003, 0.011]), and the independent indirect effect of future confidence was 0.005 (95% CI = [0.003, 0.007]). Furthermore, the chain mediating effect of life satisfaction and future confidence was 0.003 (95% CI = [0.001, 0.005]).

**Conclusion:**

Sports participation, as a positive health-related behavior, was not only directly associated with stronger fertility intentions among young adults but was also indirectly associated with fertility intentions through the independent and serial pathways involving life satisfaction and confidence in the future. These findings suggest that public sports participation and psychological well-being promotion may be considered within a broader framework of reproductive health and family-support policies. Integrating such approaches with efforts to support young adults’ physical and psychological well-being and positive expectations for the future may contribute to a more supportive social environment for family formation.

## 1 Introduction

In recent years, China has undergone profound demographic changes. Data from the Seventh National Population Census indicate that China’s total fertility rate declined to 1.3 in 2020 (1), while individuals aged 60 years and older accounted for 18.7% of the population and those aged 65 years and older accounted for 13.5%, highlighting the growing challenges of low fertility and population aging (2). To promote long-term balanced population development, China has successively implemented the “selective two-child,” “universal two-child,” and “three-child” policies, accompanied by measures such as extended maternity leave, childcare subsidies, reductions in education-related burdens, and housing support. Nevertheless, fertility intentions have not shown a substantial rebound (3), and the first-order total fertility rate has declined from approximately 0.7 to 0.5 (4). China also experienced negative population growth in both 2022 and 2023. Low fertility, therefore, is not solely a demographic issue but is also closely related to family well-being, population health, social security, and social sustainability.

From a public health perspective, fertility intentions are embedded in a complex system shaped by the interplay of economic, social, health-related, psychological, and institutional factors (5, 6). Contemporary public health research emphasizes that population health and well-being depend not only on healthcare services but also on lifestyles, psychological resources, social relationships, environmental conditions, and public policies. The combined implementation of preventive interventions, healthy lifestyles, improved living conditions, and supportive public policies is associated with multidimensional aspects of population health and quality of life (7). Psychological resilience, social cohesion, cultural continuity, and collective support are also important components of mental health and community well-being (8). Moreover, population health research suggests that health outcomes are jointly shaped by socioeconomic conditions, healthcare services, lifestyles, and preventive policies, underscoring the need for integrated health-promotion approaches (9). These perspectives suggest that fertility intentions among young adults may be related not only to economic costs but also to health status, current life experiences, and expectations about the future.

Although the traditional cost–utility perspective in family economics explains fertility-related decisions from the standpoint of economic rationality (10), it primarily emphasizes material resources and gives less attention to the multidimensional health and psychological resources involved in raising children. Psychological capital theory highlights the roles of hope, self-efficacy, resilience, and optimism in coping with uncertainty (11). The Health Promotion Model conceptualizes health behavior as being associated with individual characteristics, perceived benefits and barriers, affective experiences, and interpersonal influences (12). The broaden-and-build theory of positive emotions further proposes that positive experiences can broaden individuals’ cognitive and behavioral repertoires and facilitate the accumulation of enduring psychological resources (13). Taken together, these theoretical perspectives suggest that fertility intentions among young adults may be associated not only with objective resource conditions but also with physical and mental well-being, current life evaluations, and psychological expectations regarding the future. Sports participation is a potentially modifiable health-promoting behavior that involves not only investment in physical health but also emotional regulation, self-efficacy, experiences of mastery, and social interaction. Previous research has shown that sports participation is closely associated with physical health, emotional well-being, quality of life, and social connectedness (14). Regular participation in sports may also be associated with lower perceived stress, more positive emotions, greater self-efficacy, and a stronger sense of control over life, which in turn are related to higher life satisfaction (15). The physical, psychological, and social resources associated with sports participation may also be related to greater confidence in the future (16). Accordingly, these resources may be relevant to how young adults evaluate future family life and fertility-related choices. Life satisfaction reflects an individual’s overall evaluation of current life circumstances, whereas confidence in the future represents positive expectations regarding future development and life stability. These two psychological resources may therefore represent distinct and sequential pathways linking sports participation with fertility intentions. However, the relationships among physical activity, health, and fertility intentions may be context-dependent (12). Under conditions of substantial socioeconomic constraints, pronounced work–family conflict, or strong cultural barriers, the positive psychosocial resources associated with sports participation may not necessarily correspond to stronger fertility intentions (17). Existing research on fertility intentions has predominantly focused on structural pressures such as income, housing, employment, and education-related costs, whereas comparatively less attention has been paid to health-promoting behaviors and their potential psychological pathways. Whether sports participation is associated with fertility intentions through life satisfaction and confidence in the future, and whether these psychological resources operate independently or sequentially, has not yet been systematically examined.

Accordingly, the present study used data from the 2018 and 2022 waves of the China Family Panel Studies (CFPS) to empirically examine the mechanisms linking sports participation to fertility intentions among Chinese young adults. Specifically, this study addressed the following questions: (1) Is sports participation positively associated with fertility intentions among young adults? (2) Do life satisfaction and confidence in the future independently mediate the association between sports participation and fertility intentions? (3) Do life satisfaction and confidence in the future form a sequential mediating pathway between sports participation and fertility intentions? By integrating health-promoting behavior, current life evaluation, and future-oriented psychological expectations within a unified analytical framework, this study seeks to broaden the public health perspective on fertility intentions among young adults and to provide empirical evidence for the development of more integrated policies supporting youth health and fertility-related well-being.

### 1.1 The association between sports participation and fertility intentions among young adults

From a public health perspective, sports participation represents an important and potentially modifiable health-promoting behavior. Its value lies not only in improving physical functioning and reducing health risks (18), but also in its close associations with positive resources such as psychological well-being, self-efficacy, and social connectedness (19). The Health Promotion Model suggests that individual health behaviors are closely related to perceived benefits, affective experiences, and interpersonal influences (12). As a positive health investment and lifestyle behavior, sports participation may be associated with fertility intentions through its links with better physical and mental well-being and the accumulation of positive psychological and social resources among young adults (20).

Previous studies have shown that regular sports participation is associated with better physical health, higher subjective well-being, and a stronger sense of control over life (21, 22). Among young adults of reproductive age, better physical and psychological well-being may be associated with more positive evaluations of their health, capacity to manage daily life, and ability to assume future responsibilities, as well as with fewer concerns about future life pressures (23). These favorable psychological conditions may also correspond to greater resilience when individuals consider the challenges associated with fertility and childrearing. Meanwhile, sustained engagement, goal attainment, and self-management in sports may be associated with stronger self-efficacy and self-regulatory capacity (24), thereby providing additional psychological resources relevant to future family life and parenting responsibilities. In addition, sports participation also serves an important social interaction function. Participation in sports can provide opportunities for social interaction, broaden social networks, and be associated with greater social support and a stronger sense of group belonging (25). Favorable social support and positive interpersonal interactions are not only related to better overall life experiences among young adults but may also be positively associated with fertility intentions (26). Taken together, the physical health, psychological resources, and social support associated with sports participation may constitute an important basis for its positive association with fertility intentions among young adults.

Based on this reasoning, the following hypothesis is proposed,

*H*1: Sports participation is significantly and positively associated with fertility intentions among young adults.

### 1.2 The mediating role of life satisfaction

Life satisfaction refers to an individual’s cognitive evaluation of their overall life circumstances and quality of life and reflects their general appraisal of their current living conditions. Previous studies have shown that higher life satisfaction is generally associated with a more positive psychological state, a stronger sense of life security, and greater subjective well-being, and these positive experiences may in turn be related to fertility intentions (27). Among young adults of reproductive age, higher life satisfaction may reflect more favorable evaluations of their current health, interpersonal relationships, and overall living environment. Such positive life experiences may be associated with more favorable attitudes toward future family life and parenting responsibilities (27). In contrast, greater life stress and lower life satisfaction may be associated with heightened concerns about future family responsibilities and life uncertainty, as well as with lower fertility intentions (28).

Sports participation is an important health-promoting behavior that is closely associated with life satisfaction. Regular physical activity is related to better physical health and physiological functioning (29), as well as lower perceived stress, more positive emotional experiences, and greater social interaction (30). According to the broaden-and-build theory of positive emotions, positive emotions can broaden individuals’ cognitive and behavioral repertoires and facilitate the accumulation of psychological and social resources over time (31). Positive emotions, experiences of achievement, and social support associated with sports participation may be related to more favorable evaluations of one’s health, interpersonal relationships, and overall life circumstances, and consequently to higher levels of life satisfaction among young adults (32). From this perspective, sports participation may be associated with higher life satisfaction through better physical and psychological well-being, more positive emotional experiences, and stronger social connectedness. In turn, higher life satisfaction may be further associated with more positive expectations regarding future family life and more favorable fertility attitudes. Life satisfaction may therefore represent an important psychological pathway linking sports participation to fertility intentions among young adults.

Accordingly, we propose the following hypothesis,

*H*2: Life satisfaction significantly mediates the association between sports participation and fertility intentions among young adults.

### 1.3 The mediating role of confidence in the future

Confidence in the future refers to an individual’s overall expectations regarding future living conditions, development opportunities, and the likelihood of achieving personal goals, based on current life circumstances and perceived capabilities (33). For young adults at the stage of family formation and childbearing, fertility and childrearing involve long-term commitments, sustained investment, and considerable uncertainty (34). Their evaluations of future employment, economic circumstances, life stability, and perceived capacity to cope with future challenges may therefore be closely associated with fertility intentions (35). Previous studies have shown that greater perceived uncertainty about the future is associated with a higher likelihood of postponing or reconsidering existing fertility plans (36), whereas more positive expectations about the future are generally associated with greater confidence in family development and stronger fertility intentions (37). Confidence in the future may therefore represent an important psychological resource associated with fertility intentions among young adults.

Sports participation may also be closely associated with confidence in the future. According to psychological capital theory, positive psychological resources such as hope, self-efficacy, resilience, and optimism are relevant to how individuals cope with stress, uncertainty, and future challenges (38, 39). Regular sports participation is associated not only with better physical health and lower levels of anxiety and stress (40), but also with experiences involving goal setting, sustained engagement, overcoming challenges, and achievement, which may be associated with greater self-efficacy, psychological resilience, and a stronger sense of control over life (41). These positive psychological resources may also be reflected in broader life domains and may be associated with more positive expectations when young adults consider career development, life transitions, and future family responsibilities (42). Accordingly, sports participation may be associated with greater confidence in the future through its links with positive psychological resources. In turn, higher confidence in the future may be associated with fewer concerns about future uncertainty and with more positive expectations regarding family development and fertility. Confidence in the future may therefore represent an important psychological pathway linking sports participation to fertility intentions among young adults.

Based on this, we propose research hypothesis,

*H*3: Confidence in the future significantly mediates the association between sports participation and fertility intentions among young adults.

### 1.4 The chain mediating role of life satisfaction and confidence in the future

Life satisfaction and confidence in the future reflect young adults’ subjective evaluations of their current lives and future development, respectively. Rather than being independent of one another, these two constructs may form a sequential psychological process extending from “current life experiences” to “future development expectations.” Life satisfaction primarily reflects an individual’s evaluation of current living conditions and overall quality of life (43), whereas confidence in the future represents positive expectations regarding future life stability, development opportunities, and one’s perceived capacity to cope with future challenges (44). Previous studies have shown that positive evaluations of current life circumstances are closely associated with more optimistic expectations about the future (45). Among young adults at the stage of family formation and childbearing, satisfaction with current life may provide an important psychological basis for evaluating future life stability and the perceived manageability of family responsibilities (46). The broaden-and-build theory of positive emotions provides a theoretical perspective for understanding this sequential process (31). According to this theory, positive emotions and favorable life experiences are associated with broader cognitive and behavioral repertoires and with the gradual accumulation of enduring psychological resources (47). Better physical health, positive emotional experiences, and social interaction associated with sports participation may first be related to higher life satisfaction among young adults (48). In turn, the sense of security, perceived gains, and favorable evaluations of life reflected in higher life satisfaction may be associated with greater hope, optimism, and perceived control over life, and consequently with stronger confidence in the future (49).

Furthermore, confidence in the future may represent a psychological link through which positive resources associated with current life experiences are reflected in longer-term family planning. Fertility and childrearing involve substantial long-term commitments and uncertainty. Young adults may therefore rely not only on positive evaluations of their current lives but also on favorable expectations regarding future life stability and their capacity to assume family responsibilities (50). From this perspective, sports participation may first be associated with higher life satisfaction, which may in turn be related to stronger confidence in future development; greater confidence in the future may then be associated with more favorable fertility attitudes. Life satisfaction and confidence in the future may therefore constitute a serial psychological pathway linking sports participation to fertility intentions, extending from “current life evaluation” to “future development expectations.”.

Accordingly, we propose the following hypothesis,

*H*4: Life satisfaction and confidence in the future serially mediate the association between sports participation and fertility intentions among young adults.

The proposed chain mediation model linking sports participation to fertility intentions is presented in Figure 1.

**Figure 1:**
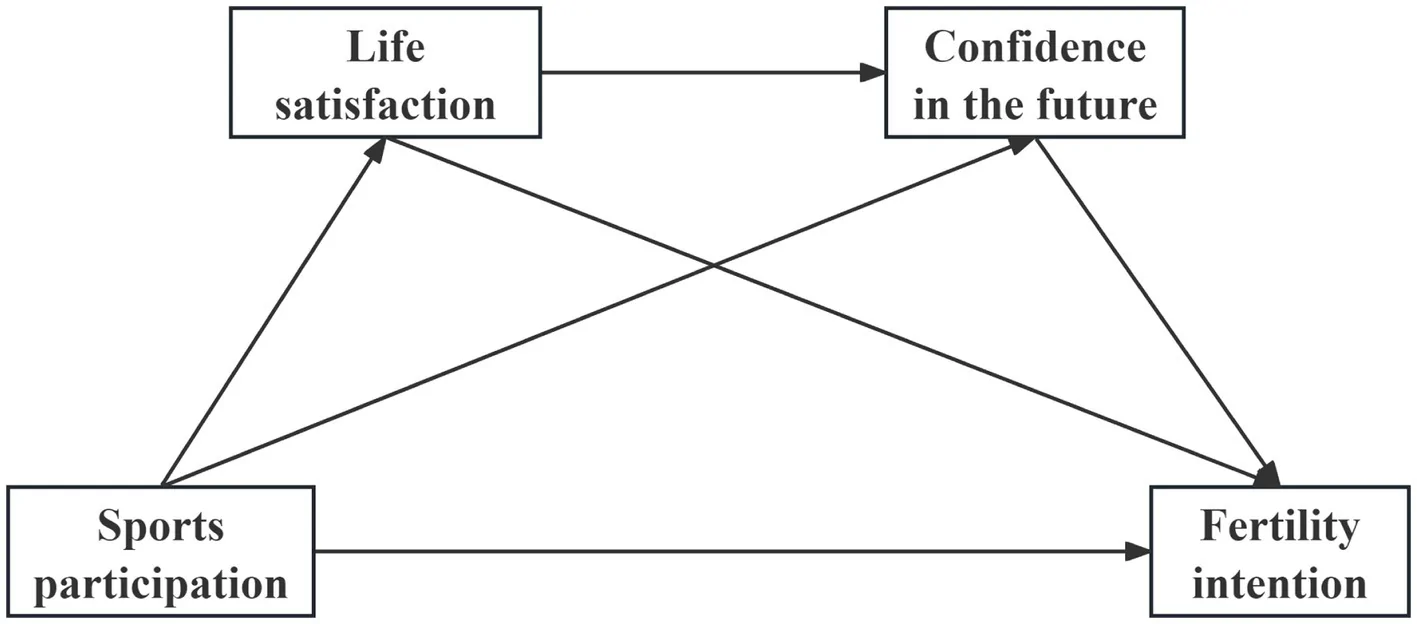
Theoretical framework of the chain mediation model.

## 2 Materials and methods

### 2.1 Data sources

The data used in this study were drawn from the 2018 and 2022 waves of the China Family Panel Studies (CFPS), conducted by the Institute of Social Science Survey at Peking University. The CFPS employs a multistage probability proportional to size sampling design to ensure sampling rigor and representativeness. The survey covers 25 provinces, autonomous regions, and municipalities in mainland China, excluding Hong Kong, Macao, Taiwan, Xinjiang, Tibet, Inner Mongolia, Ningxia, Qinghai, and Hainan. The covered population accounts for approximately 95% of the total population of mainland China, providing broad national representativeness. Given evidence that fertility intentions changed among some population groups following the implementation of China’s universal two-child policy in 2016 (51), data from the 2018 and 2022 waves were selected for the present analysis. Following previous studies of fertility intentions using CFPS data, the analytical sample was restricted to men aged 22–45 years and women aged 20–40 years (20). The lower age limits correspond to the legal minimum age for marriage in China—22 years for men and 20 years for women—whereas the upper age limits were based on the same sex-specific age criteria adopted in previous fertility research. Among the 14,634 observations meeting the study inclusion criteria, the proportion of missing data for individual study variables was generally low, ranging from 0 to 4.81%. Specifically, sports participation was missing for 704 observations (4.81%), fertility intentions for 669 observations (4.57%), life satisfaction for 652 observations (4.46%), confidence in the future for 617 observations (4.22%), and sex for 598 observations (4.09%); the proportion of missing data for all other variables was below 4%. Across all study variables, 926 observations (6.33%) had missing data on at least one variable and were therefore excluded from the complete-case analysis, leaving 13,708 observations (93.67%) for the main analyses. To further assess the missing-data mechanism, Little’s test of Missing Completely at Random (MCAR) was conducted. The result was not statistically significant, χ^2^(158) = 183.56, *p* = 0.080, indicating that the null hypothesis of MCAR could not be rejected and that there was no statistical evidence that the observed missing-data pattern significantly deviated from the MCAR assumption.

### 2.2 Variable selection

#### 2.2.1 Fertility intentions

Following previous studies (52, 53), fertility intention was measured using the CFPS question, “How many children would you like to have?” This indicator captures the respondent’s desired number of children and is therefore a count-type variable rather than an ordinal Likert-scale measure of attitudinal intensity. Because relatively few respondents reported a desired number of children greater than four, all values above four were top-coded as four to reduce the influence of extreme observations and improve the interpretability of the estimates. The final fertility intention variable therefore ranged from 0 to 4, with higher values indicating a greater desired number of children.

#### 2.2.2 Sports participation

In alignment with established research (20), sports participation was operationalized as a binary variable coded 0 or 1. The 2018 and 2022 CFPS waves differed substantially in the reference periods and response categories used to measure exercise frequency. In 2018, respondents were asked, “How many times did you participate in physical exercise during the past week?”, with responses ranging from 0 to 50. In 2022, respondents were asked to report their average frequency of physical exercise during the previous 12 months using eight response categories. Because the original frequency measures from the two waves were not directly comparable, we harmonized them using a common criterion of whether respondents participated in physical exercise at least once per week on average, thereby improving comparability across waves. Specifically, in the 2018 data, respondents who reported no physical exercise during the previous week were coded as 0, whereas those who reported exercising at least once were coded as 1. In the 2022 data, respondents who selected “less than once per month on average,” “at least once per month but less than once per week,” or “never” were classified as exercising less than once per week and coded as 0. Those who selected “1–2 times per week on average,” “3–4 times per week on average,” “5 or more times per week on average,” “once per day,” or “twice or more per day” were classified as exercising at least once per week and coded as 1. This variable therefore captures whether respondents engaged in regular weekly physical exercise rather than their specific exercise frequency, session duration, or intensity. Although this binary harmonization helps reduce measurement discrepancies arising from differences in questionnaire design across the two waves, it also entails some loss of information regarding the dose of physical activity.

#### 2.2.3 Life satisfaction

In alignment with established research (54), life satisfaction was captured through the question, “How satisfied are you with your life?” in the CFPS questionnaire. This item was assessed utilizing a 5-point Likert scale, ranging from 1 (“very dissatisfied”) to 5 (“very satisfied”). Higher operational scores on this metric correspond to elevated configurations of subjective life satisfaction.

#### 2.2.4 Confidence in the future

In alignment with established research (55), confidence in the future was captured through the question, “How confident are you about your future?” in the CFPS questionnaire. This item was assessed utilizing a 5-point Likert scale, ranging from 1 (“not confident at all”) to 5 (“very confident”). Higher operational scores on this metric correspond to elevated configurations of subjective confidence in the future.

#### 2.2.5 Control variables

The control variables included sex, age, hukou status, educational attainment, marital status, self-rated health, household income, and other relevant covariates. Annual per capita household income was transformed using the natural logarithm of income plus one [ln(income + 1)]. Adding one before logarithmic transformation allowed observations with an original income value of zero to be retained. Accordingly, the minimum value of 0 for the transformed income variable corresponds to an original income of zero rather than an annual income of 1 yuan. It should also be noted that sex was used primarily for descriptive statistics and heterogeneity analyses. Because sex is time-invariant at the individual level, it is perfectly collinear with the individual fixed effects and is therefore absorbed by them. Accordingly, its coefficient was not separately estimated in either the baseline fixed-effects models or the fixed-effects mediation models. Detailed descriptive statistics and operational definitions for the primary variables are presented in Table 1.

**Table 1:** Variable definitions and descriptive statistics.

Variable type	Variables	Variable declaration	Mean	SD	Min	Max
Dependent variable	Fertility intention	The variable ranged from 0 to 4 and represented the respondent’s desired number of children: 0 = no children, 1 = one child, 2 = two children, 3 = three children, and 4 = four or more children.	1.884	0.477	0	4
Independent variable	Sports participation	0 = No participation; 1 = Participation	0.341	0.473	0	1
Mediating variable	Life satisfaction	Ranges from 1 to 5, where 1 indicates “very dissatisfied” and 5 indicates “very satisfied.”	3.962	0.776	1	5
	Confidence in the future	Ranges from 1 to 5, where 1 indicates “not confident at all” and 5 indicates “very confident.”	4.185	0.730	1	5
Control variables	Gender	0 = Female; 1 = Male	0.522	0.499	0	1
	Age	Actual age of respondents at the time of the survey	32.218	6.259	20	45
	Hukou status	0 = Agricultural hukou; 1 = Non-agricultural hukou	0.256	0.436	0	1
	Education	1 = Nursery school; 2 = kindergarten/preschool class; 6 = Primary school; 9 = Junior high school; 12 = Senior high school, including secondary vocational school; 15 = Junior college; 16 = Bachelor’s degree; 19 = Master’s degree	8.406	4.289	1	19
	Marital status	0 = Unmarried; 1 = Married	0.764	0.424	0	1
	Self-rated health	1 = Very unhealthy; 2 = Relatively unhealthy; 3 = Fair; 4 = Relatively healthy; 5 = Very healthy	3.382	0.995	1	5
	Income	Natural logarithm of annual per capita household income plus one, ln(income + 1).	8.008	4.454	0	14.40
	Subjective social status	Ranges from 1 to 5, where 1 indicates “very low” and 5 indicates “very high.”	2.863	0.924	1	5

### 2.3 Statistical analysis

Data cleaning and statistical analyses were performed using Stata 18.0. First, a two-way fixed-effects model was constructed to examine the association between sports participation and fertility intentions among young adults (Hypothesis 1), while controlling for both individual and year fixed effects. The model was specified as follows:


$$\text{Fertilit}y_{\mathrm{it}}=\beta_{0}+\beta_{1}\text{Sport}s_{\mathrm{it}}+\gamma C_{\mathrm{it}}+\mu_{i}+\lambda_{t}+\varepsilon_{\mathrm{it}}$$

$\text{Fertilit}y_{\mathrm{it}}$ represents the fertility intention of individual i in survey year t ; $\text{Sport}s_{\mathrm{it}}$ denotes the sports participation; $C_{\mathrm{it}}$ represents the control variables; $\mu_{i}$ indicates the individual fixed effects; $\lambda_{t}$ represents the year fixed effects; $\varepsilon_{\mathrm{it}}$ is the random error term; $\beta_{0}$ represents the intercept; $\beta_{1}$ and γ denote the coefficients for sports participation and the control variables, respectively.

Second, to examine the potential psychological pathways linking sports participation to fertility intentions among young adults, we developed a customized program in Stata that combined individual-level cluster bootstrap resampling with individual fixed-effects regression. Using 5,000 bootstrap resamples, we separately tested the mediating role of life satisfaction (Hypothesis 2), the mediating role of confidence in the future (Hypothesis 3), and the serial mediating role of life satisfaction and confidence in the future (Hypothesis 4). Before model estimation, multicollinearity and model diagnostic tests were conducted. The variance inflation factor (VIF) values for the variables included in the model ranged from 1.08 to 1.77, all below the threshold of 5, with a mean VIF of 1.36, indicating no evidence of serious multicollinearity. The residual-versus-fitted plot showed that the LOWESS smoothed curve remained close to the zero reference line across most of the fitted-value range and did not exhibit a pronounced systematic curvilinear pattern, providing no clear evidence of substantial nonlinearity in the model specification. The normal Q–Q plot showed a stepwise pattern in the residuals, with deviations from the reference line in some regions and in the tails; the joint skewness–kurtosis test further rejected the null hypothesis of normally distributed residuals, χ^2^(2) = 421.53, *p* < 0.001. In addition, the Breusch–Pagan/Cook–Weisberg test rejected the null hypothesis of constant error variance, χ^2^(1) = 484.71, *p* < 0.001, indicating the presence of heteroskedasticity. Given the panel structure of the data and the potential for heteroskedasticity and within-individual dependence arising from repeated observations, individual-level cluster-robust standard errors were used for statistical inference. Detailed model diagnostic results are provided in the Supplementary material.

## 3 Results

### 3.1 The association between sports participation and fertility intentions among young adults

A two-way fixed-effects model was estimated with fertility intentions as the dependent variable and sports participation as the primary independent variable, while adjusting for age, hukou status, educational attainment, marital status, self-rated health, household income, and subjective social status. The results are presented in Table 2. Model 1 included only the control variables. The results showed that hukou status, educational attainment, and household income were significantly negatively associated with fertility intentions, whereas marital status, self-rated health, and subjective social status were significantly positively associated with fertility intentions. Building upon Model 1, Model 2 incorporated the sports participation variable. After accounting for individual and year fixed effects alongside other control variables, sports participation exhibited a significant positive association with youths’ fertility intention (*β* = 0.046, *p* < 0.01). Specifically, within the same individual, periods of sports participation were associated with fertility-intention scores that were, on average, 0.046 units higher than periods of non-participation, indicating that within-person changes in sports participation status were positively associated with changes in fertility intentions. Thus, Hypothesis 1 was supported.

**Table 2:** Baseline regression results.

Variables	Model 1 (Fertility intention)	Model 2 (Fertility intention)
Sports participation		0.046^**^(0.015)
Age	−0.024(0.044)	−0.031(0.044)
Hukou status	−0.112^***^(0.010)	−0.124^***^(0.010)
Education	−0.018^***^(0.001)	−0.021^***^(0.001)
Marital status	0.156^***^(0.011)	0.169^***^(0.011)
Self-rated health	0.026^***^(0.004)	0.023^***^(0.004)
Income	−0.007^***^(0.002)	−0.006^***^(0.002)
Subjective social status	0.036^***^(0.004)	0.033^***^(0.004)
Individual fixed effects	Yes	Yes
Year fixed effects	Yes	Yes
Constant	2.772^***^(0.233)	2.981^***^(0.250)
Observations	13,708	13,708

*p < 0.05, **p < 0.01, ***p < 0.001.

In terms of effect magnitude, the regression coefficient for sports participation was 0.046, indicating a relatively small absolute association compared with the 0–4 range of the fertility-intention variable. Accordingly, this finding should not be interpreted as suggesting that sports participation substantially increases fertility intentions among young adults. Rather, it indicates a modest but statistically robust positive association between sports participation and fertility intentions. Given that fertility intentions are shaped by multiple economic, familial, institutional, and sociocultural factors, even a relatively small average association may still have relevance for public health and population policy when considered across a large population of young adults.

### 3.2 Robustness checks

To enhance the credibility of the findings, several robustness checks were conducted. First, because the CFPS data used in this study included only two survey waves, 2018 and 2022, individual fixed effects could no longer be identified after introducing a one-period lagged explanatory variable. Therefore, Model 3 employed a cross-sectional ordinary least squares regression using the 2022 data. Sports participation in 2018 was included as the lagged explanatory variable, while baseline fertility intentions in 2018 and the other control variables were also adjusted for. As shown in Model 3 of Table 3, lagged sports participation was significantly and positively associated with subsequent fertility intentions, with a coefficient of 0.068 (*p* < 0.001). After adjustment for baseline fertility intentions and the other covariates, respondents who participated in sports in the earlier wave reported higher fertility intentions in the subsequent wave. The direction of this association was consistent with that observed in the baseline regression, providing additional evidence of a temporally lagged association between sports participation and fertility intentions.

**Table 3:** Results of the robustness checks.

Variables	Model 3 (Fertility intention)	Model 4 (Fertility intention)	Model 5 (Fertility intention)
Sports participation			0.071^***^(0.005)
Duration of each exercise session (reference: no sports participation)			
1–30 min		0.074^***^(0.020)	
31–60 min		0.088^***^(0.021)	
≥ 61 min		0.169^***^(0.028)	
Lagged sports participation	0.068^***^(0.015)		
Baseline fertility intention	0.477^***^(0.025)		
Gender	0.058^***^(0.015)	0.119^***^(0.016)	
Age	0.003(0.002)	0.002^*^(0.001)	0.002^***^(0.000)
Hukou status	−0.093^***^(0.020)	−0.185^***^(0.017)	−0.074^***^(0.006)
Education	−0.009^***^(0.002)	−0.015^***^(0.002)	−0.011^***^(0.001)
Marital status	0.179^***^(0.026)	0.317^***^(0.021)	0.104^***^(0.007)
Self-rated health	0.010(0.007)	−0.012(0.008)	0.015^***^(0.002)
Income	−0.006^**^(0.002)	−0.002(0.002)	−0.001(0.000)
Subjective social status	0.020^*^(0.008)	0.031^***^(0.009)	0.019^***^(0.002)
Constant	0.755^***^(0.079)	1.550^***^(0.056)	0.471^***^(0.018)
$R^{2}$	0.316	0.163	—

*p < 0.05, **p < 0.01, ***p < 0.001.

Second, a robustness analysis was conducted using the 2022 CFPS data by replacing the binary indicator of sports participation with duration per exercise session. Following previous studies, exercise duration per session was categorized into four groups: 0 min (no sports participation), 1–30 min, 31–60 min, and ≥61 min. This analysis was used to examine whether fertility intentions differed across categories of exercise duration. As shown in Model 4 of Table 3, compared with respondents who did not participate in sports, those reporting exercise durations of 1–30 min, 31–60 min, and ≥61 min per session had significantly higher fertility intentions, with regression coefficients of 0.074, 0.088, and 0.169, respectively (all *p* < 0.001). The magnitude of the positive association generally increased across the exercise-duration categories, with the largest association observed in the ≥61 min group. Although these findings should not be interpreted as evidence of a causal dose–response relationship, they suggest that the baseline results were not solely attributable to the binary operationalization of sports participation. Third, a fixed-effects Poisson model was estimated as an additional sensitivity analysis. As shown in Model 5 of Table 3, sports participation remained significantly and positively associated with fertility intentions (*β* = 0.071, *p* < 0.001). The direction of the association was consistent with that obtained from the two-way fixed-effects linear model, further supporting the robustness of the main findings.

### 3.3 Testing the chain mediation effect

To make full use of the two-wave panel data and account for within-individual dependence arising from repeated observations of the same respondents, a custom Stata program was developed to examine the serial mediation effects. An individual-level cluster bootstrap procedure was combined with fixed-effects regressions, with 5,000 bootstrap replications. In each replication, respondents were resampled at the individual level, and all available observations for each selected respondent were retained, thereby reducing the risk of underestimated standard errors due to within-individual serial correlation. The models adjusted for age, hukou status, marital status, self-rated health, household income, and subjective social status, while the individual fixed effects absorbed sex and other observed and unobserved time-invariant individual characteristics. The statistical significance of the mediation effects was assessed using bias-corrected 95% confidence intervals.

The results of the chain mediation analysis are presented in Table 4. The estimated total effect of sports participation on fertility intentions among young adults was 0.049 (95% CI [0.021, 0.077]). After introducing life satisfaction and confidence in the future as mediators, the direct effect of sports participation on fertility intentions was 0.034 (95% CI [0.010, 0.058]). The total indirect effect through life satisfaction and confidence in the future was 0.015 (95% CI [0.009, 0.020]), accounting for 30.61% of the total effect. Specifically, the indirect effect through life satisfaction was 0.007 (95% CI [0.003, 0.011]), accounting for 14.29% of the total effect, thus supporting Hypothesis 2. The indirect effect through confidence in the future was 0.005 (95% CI [0.003, 0.007]), accounting for 10.20% of the total effect, thus supporting Hypothesis 3. Furthermore, the chain mediation effect through life satisfaction and confidence in the future was 0.003 (95% CI [0.001, 0.005]), supporting Hypothesis 4. The chain mediation model is illustrated in Figure 2.

**Table 4:** Bootstrap test results for the chain mediation model.

Path	Effect size	Boot SE	Boot 95% CI LLCI	Boot 95% CI ULCI	Percentage (%)
Total effect	0.049	0.014	0.021	0.077	100%
Direct effect	0.034	0.012	0.010	0.058	69.39%
Total indirect effect	0.015	0.002	0.009	0.020	30.61%
Sports participation → life satisfaction → fertility intention	0.007	0.002	0.003	0.011	14.29%
Sports participation → confidence in the future → fertility intention	0.005	0.001	0.003	0.007	10.20%
Sports participation → life satisfaction → confidence in the future → fertility intention	0.003	0.0009	0.001	0.005	6.12%

**Figure 2:**
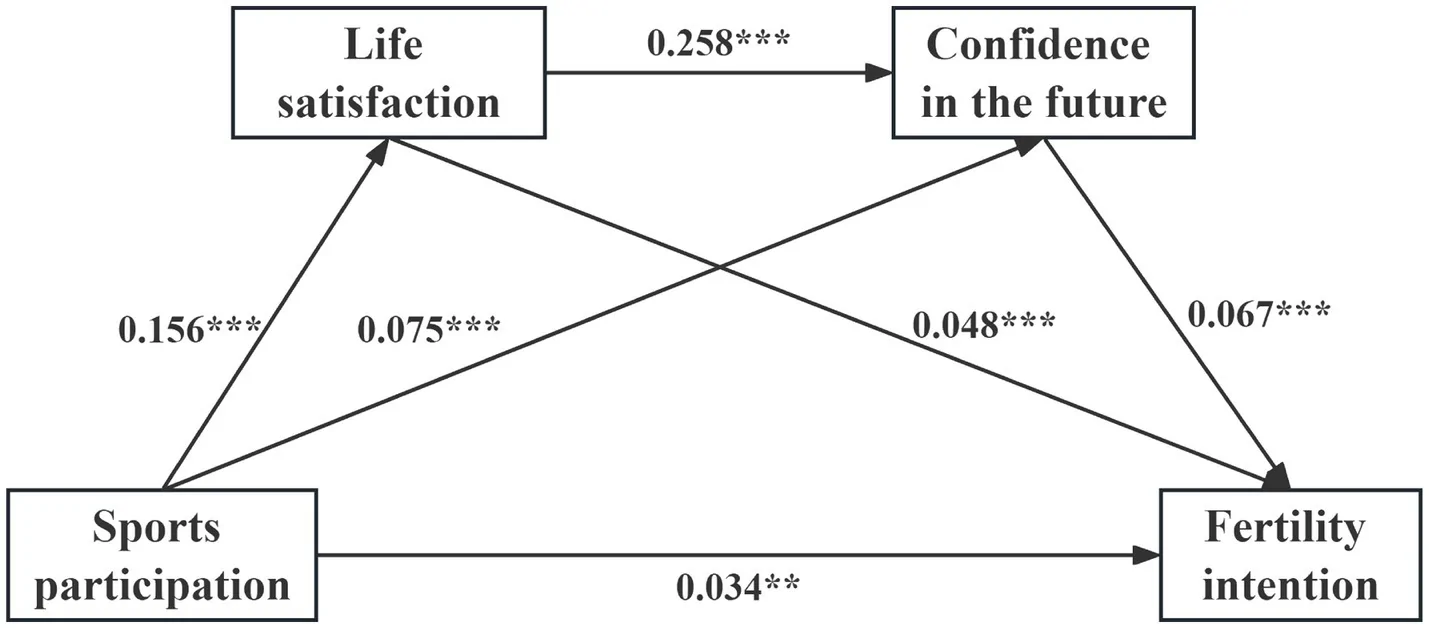
Chain mediation model of sports participation and fertility intention. *p < 0.05, **p < 0.01, ***p < 0.001.

In terms of the relative contributions of the direct and indirect effects, the direct effect accounted for 69.39% of the total effect, whereas the total indirect effect associated with the psychological pathways accounted for 30.61%. Among the indirect pathways, the pathway through life satisfaction alone accounted for approximately 46.67% of the total indirect association, the pathway through confidence in the future alone accounted for approximately 33.33%, and the serial pathway through life satisfaction followed by confidence in the future accounted for approximately 20.00%. Thus, life satisfaction represented the largest single indirect pathway in relative terms, whereas the serial pathway had the smallest absolute magnitude, although it remained statistically significant. Taken together, these findings suggest that the association between sports participation and fertility intentions among young adults is reflected not only in a direct statistical association but also in indirect associations involving psychological resources such as positive evaluations of current life and expectations about the future.

### 3.4 Heterogeneity analysis

To further examine whether the association between sports participation and fertility intentions varied across sociodemographic groups, complementary random-effects models with interaction terms were estimated for sex, hukou status, educational attainment, and marital status. Unlike the baseline individual fixed-effects models, which primarily rely on within-individual variation, the heterogeneity analyses were intended to compare differences in the sports participation–fertility intention association across population groups and therefore retained both within- and between-individual variation in these sociodemographic characteristics. The results of the heterogeneity analyses are presented in Table 5.

**Table 5:** Results of the heterogeneity analysis.

Variables	Fertility intention			
	Model 6 (Gender)	Model 7 (Hukou Status)	Model 8 (Education)	Model 9 (Marital Status)
Sports participation	0.102^***^(0.012)	0.107^***^(0.010)	0.114^***^(0.008)	0.197^***^(0.016)
Gender	0.014(0.011)			
Sports participation × gender	0.031^*^(0.016)			
Hukou status		−0.141^***^(0.013)		
Sports participation × hukou status		0.040^*^(0.017)		
Education			−0.023^***^(0.001)	
Sports participation × education			0.005^**^(0.002)	
Marital status				0.215^***^(0.013)
Sports participation × marital status				−0.105^***^(0.018)
Control variables	Included	Included	Included	Included
Constant	1.641^***^(0.032)	1.640^***^(0.032)	1.461^***^(0.029)	1.593^***^(0.032)

*p < 0.05, **p < 0.01, ***p < 0.001.

Model 6 examined heterogeneity by gender. Sports participation was significantly and positively associated with fertility intentions (*β* = 0.102, *p* < 0.001). The interaction term between sports participation and sex was significantly positive (*β* = 0.031, *p* < 0.05), indicating that the association between sports participation and fertility intentions differed significantly by sex, with a stronger positive association observed among men than among women. Model 7 examined heterogeneity by hukou status. Sports participation was significantly and positively associated with fertility intentions (*β* = 0.107, *p* < 0.001). The interaction term between sports participation and hukou status was significantly positive (*β* = 0.040, *p* < 0.05), indicating a significant difference between young adults with agricultural and non-agricultural hukou. Specifically, the positive association between sports participation and fertility intentions was more pronounced among those with non-agricultural hukou. Model 8 further examined heterogeneity by educational attainment. When educational attainment was at the sample mean, sports participation was significantly and positively associated with fertility intentions (*β* = 0.114, *p* < 0.001). The interaction term between sports participation and educational attainment was significantly positive (*β* = 0.005, *p* < 0.01), suggesting that the positive association between sports participation and fertility intentions became stronger as educational attainment increased. Model 9 examined heterogeneity by marital status. Sports participation was significantly and positively associated with fertility intentions (*β* = 0.197, *p* < 0.001). The interaction term between sports participation and marital status was significantly negative (*β* = −0.105, *p* < 0.001), indicating that the association between sports participation and fertility intentions differed significantly between unmarried and married young adults. Specifically, the positive association was substantially stronger among unmarried young adults than among their married counterparts.

## 4 Discussion

### 4.1 The association between sports participation and fertility intentions among young adults

The present study found a significant positive association between sports participation and fertility intentions among young adults. In the baseline model, the regression coefficient for sports participation was 0.046, indicating that, after accounting for individual and year fixed effects, within-person changes in sports participation status were positively associated with changes in fertility intentions. Consistent with previous findings from China on the relationship between sports participation and fertility intentions (20, 56), the present results suggest that sports participation is associated not only with physical and psychological well-being but also with young adults’ longer-term life planning and intentions regarding family formation. Health-related behaviors may therefore provide a complementary public health perspective for understanding variation in fertility intentions among young adults.

At the same time, the absolute magnitude of the coefficient for sports participation was relatively small (*β* = 0.046), and sports participation should therefore not be regarded as a central determinant of fertility intentions. Fertility intentions are associated with a complex combination of structural, familial, and individual factors (57), whereas sports participation may be more appropriately understood as a health-related behavior associated with physical health, psychological well-being, and overall quality of life (58). In other words, the significance of the present study does not lie in suggesting that sports participation could directly address low fertility, but rather in highlighting the broader connections between fertility intentions, health behaviors, and overall well-being among young adults (5). The physical, psychological, and social dimensions of sports participation provide a plausible basis for understanding this association. Previous studies have shown that regular sports participation is associated with better physical health, lower psychological stress, greater social interaction, and more positive experiences (18, 25). Among young adults at the stage of family formation and childbearing, these experiences may be related to more favorable evaluations of their own health, ability to manage daily life, and capacity to assume future family responsibilities (25). Particularly in contexts characterized by substantial uncertainty about the future, favorable physical and psychological well-being and stronger social support may represent important personal resources relevant to how young adults evaluate future family life and may therefore be positively associated with fertility intentions.

It is important to note that studies directly examining the association between sports participation and fertility intentions remain limited, with most available empirical evidence derived from the Chinese context (20). In contrast, international research has focused more extensively on the relationships between physical activity and fecundity, reproductive health, and assisted reproductive outcomes. Existing evidence suggests that these relationships do not follow a uniformly linear pattern and may vary according to physical activity intensity, body weight status, and population characteristics. For example, Wise et al. reported that moderate physical activity was associated with higher fecundability among women, whereas higher levels of vigorous activity were associated with a longer time to pregnancy; however, the latter association was less evident among women with overweight or obesity (59). McKinnon et al. similarly found that moderate physical activity was associated with higher fecundability among women overall, whereas the positive association between vigorous activity and fecundability was mainly observed among women with overweight or obesity (60). Findings from studies of assisted reproduction have likewise been inconsistent. Some studies found no significant associations between physical activity and embryo implantation, clinical pregnancy, or live birth (61). An earlier meta-analysis reported that physical activity was associated with higher rates of clinical pregnancy and live birth, whereas associations with implantation and miscarriage rates were not statistically significant (62). A more recent systematic review and meta-analysis further indicated that physical activity was positively associated with clinical pregnancy rates, while evidence regarding live birth, miscarriage, and embryo implantation remained uncertain, with substantial heterogeneity across studies (63).

Taken together, these findings suggest that the relationships between physical activity and fertility-related outcomes may depend on activity intensity, population characteristics, and contextual conditions. It would therefore be inappropriate to assume that sports participation is uniformly associated with more favorable fertility-related outcomes. Given the limited cross-national evidence directly examining sports participation and fertility intentions, the positive association observed in the present study should be interpreted within China’s specific sociocultural context, family system, and fertility-support environment. Whether this association is stable across different cultural and institutional settings remains to be examined in future research.

### 4.2 The mediating role of life satisfaction

The findings indicated that life satisfaction significantly mediated the association between sports participation and fertility intentions among young adults. Specifically, sports participation was significantly associated with higher life satisfaction, which in turn was associated with stronger fertility intentions, suggesting that life satisfaction may represent an important psychological pathway linking sports participation with fertility intentions. This finding further suggests that fertility intentions among young adults are related not only to external conditions such as income, housing pressure, marital status, and policy support, but also to individuals’ subjective evaluations of their current lives (46, 64). First, regular physical activity has been associated with better physical health, lower levels of stress, more positive emotional experiences, and greater subjective well-being (65, 66). Second, the social interaction associated with sports participation may provide more opportunities for interpersonal engagement and is related to greater social support and lower levels of loneliness (67), which are, in turn, positively associated with life satisfaction. Higher life satisfaction may also reflect more favorable evaluations of current life stability and of the psychological and social resources available to the individual. According to the broaden-and-build theory, positive emotions and favorable life experiences are associated with broader cognitive and behavioral repertoires and with the accumulation of enduring psychological resources (68). Among young adults at the stage of family formation and childbearing, more positive evaluations of current life may be associated with lower fertility-related anxiety and a greater sense of life security (46, 69), and may consequently be related to stronger fertility intentions.

It is worth noting that the specific indirect effect through life satisfaction accounted for only 14.29% of the total effect, indicating that life satisfaction explained only part of the overall association between sports participation and fertility intentions. Other factors, such as relationship quality, social support networks, and family values, may also be relevant. Life satisfaction should therefore not be regarded as the sole psychological mechanism underlying this association. From a public health perspective, life satisfaction itself is an important indicator of psychological well-being and overall population well-being among young adults. These findings suggest that the development of a fertility-friendly social environment should not focus exclusively on material incentives, but should also consider broader efforts to support young adults’ quality of life and psychological well-being.

### 4.3 The mediating role of future confidence

The findings indicated a significant mediating pathway involving confidence in the future in the association between sports participation and fertility intentions among young adults. Sports participation was associated with greater confidence in the future, which in turn was associated with stronger fertility intentions, suggesting that confidence in the future may represent an important psychological link between current health-related behaviors and longer-term expectations regarding family formation. Fertility intentions are inherently future-oriented, reflecting not only current living conditions but also individuals’ expectations regarding future economic circumstances, the broader social environment, and life stability (37). The association between sports participation and confidence in the future may be related to experiences of goal attainment, competence, and perceived control that accompany sports participation (70). Sports activities typically involve sustained effort, coping with challenges, and achieving incremental goals, and experiences of competence and mastery in sports settings have been closely associated with self-efficacy and psychological resilience (71, 72). Previous research has also found that regular exercise is associated with higher self-efficacy and more positive self-evaluations among young adults (73), while greater self-efficacy and resilience are closely related to individuals’ perceived capacity to cope with life stressors and challenges (74). Accordingly, the positive psychological resources associated with sports participation may extend beyond the exercise context and be related to young adults’ confidence in their ability to manage future life challenges, providing one possible explanation for the positive association between sports participation and confidence in the future. At the same time, Confidence in the future is also theoretically relevant to fertility intentions. Childbearing involves long-term economic commitments, caregiving responsibilities, and family obligations; therefore, perceptions of future stability and predictability, as well as confidence in one’s capacity to cope with potential risks, may represent important psychological considerations in fertility intentions. Previous studies have shown that expectations regarding future economic and social conditions are closely associated with fertility intentions (37), while greater social and employment uncertainty tends to be associated with lower or more uncertain fertility intentions (23). Individuals’ perceived capacity to cope with future economic shocks has likewise been associated with fertility intentions (75). More recent evidence further suggests that uncertainty regarding one’s ability to cope with future employment loss is associated with lower fertility intentions (76). These findings suggest that young adults’ perceptions of future life stability and their capacity to manage uncertainty may represent an important psychological dimension of fertility intentions, providing theoretical support for the observed mediating role of confidence in the future.

From a public health perspective, sports participation and mental health promotion may form part of broader efforts to support the overall well-being of young adults, but they should not be regarded as stand-alone approaches to promoting fertility. Rather, health promotion may be more appropriately integrated with policies addressing employment, housing, childcare, reproductive health services, and work–family balance, with the broader goal of supporting young adults’ current quality of life and more positive expectations regarding their future living conditions.

### 4.4 The chain mediating effect of life satisfaction and future confidence

The results further showed a significant serial mediating pathway involving life satisfaction and confidence in the future in the association between sports participation and fertility intentions among young adults. Specifically, sports participation was associated with higher life satisfaction, which was in turn associated with greater confidence in the future, and greater confidence in the future was further associated with stronger fertility intentions. This finding suggests that the association between sports participation and fertility intentions may involve a sequential psychological process extending from evaluations of current life circumstances to expectations about future development (5). Life satisfaction and confidence in the future represent two distinct but interrelated temporal dimensions. Life satisfaction primarily reflects an individual’s cognitive evaluation of overall quality of life (77), whereas confidence in the future more closely reflects positive expectations regarding future development and life possibilities (78). Previous studies have also reported a positive association between life satisfaction and future expectations (79), while future expectations have been closely linked to fertility plans among young adults (27, 37). Sports participation may represent an important correlate within this sequential psychological pathway. Previous research has associated sports participation with higher life satisfaction, self-esteem, psychological well-being, and more positive social relationships (25). These positive life experiences may be related to more favorable evaluations of current life circumstances and, in turn, to greater confidence in the future. Fertility intentions are also inherently future-oriented, and previous studies have shown that expectations regarding future economic and social conditions are closely associated with fertility intentions (37). In addition, cross-national longitudinal evidence has linked higher life satisfaction with a greater subsequent likelihood of childbearing, further highlighting the relevance of subjective life evaluations to family formation (27). Taken together, the serial mediation observed in this study may reflect a potential psychological pathway extending from current life evaluations to future expectations and, subsequently, to longer-term intentions regarding family formation.

From a public health perspective, these findings suggest that understanding fertility intentions among young adults requires attention not only to current living conditions but also to subjective evaluations of life and expectations about the future. Both life satisfaction and confidence in the future are important dimensions of psychological well-being and social welfare and may be related to broader social conditions, including employment, housing, social support, and family environments. Sports participation may therefore be more appropriately considered within a broader framework for promoting youth health and well-being, alongside mental health services, employment support, housing assistance, childcare provision, and family-friendly policies. Such an integrated approach may better support young adults’ current quality of life and their expectations regarding future living conditions, rather than treating sports participation as a stand-alone policy instrument for increasing fertility intentions.

### 4.5 Heterogeneity in the association between sports participation and fertility intentions

The heterogeneity analyses showed that the positive association between sports participation and fertility intentions was more pronounced among men, young adults with non-agricultural hukou, those with higher educational attainment, and unmarried young adults. These differences suggest that the association may vary according to social roles, stages of family formation, and broader resource environments.

First, with respect to sex, the observed difference may be related to the different role expectations and practical burdens faced by men and women during family formation. Previous studies have shown that women generally assume a greater share of housework and childcare responsibilities and experience more pronounced work–family conflict, and these structural pressures are associated with lower fertility intentions (80, 81). Evidence from China similarly suggests that traditional gender roles, women’s greater caregiving responsibilities, and workplace pressures may represent important contextual factors underlying gender differences in fertility intentions (5). Accordingly, the physical and psychological health resources associated with sports participation may be more constrained by family responsibilities and structural pressures among women, whereas men’s relatively lower direct caregiving burden may be consistent with the stronger positive association observed among men.

Second, with respect to marital status, the positive association between sports participation and fertility intentions was more pronounced among unmarried young adults. Marital and partnership status represents an important life-course condition in the process of family formation, and fertility intentions may vary with partnership experiences, career development, and previous reproductive experiences (82, 83). Among married young adults, fertility intentions may be more closely related to concrete circumstances such as childcare costs, housing conditions, employment, and caregiving arrangements (84). In contrast, fertility intentions among unmarried young adults may more strongly reflect expectations regarding future family formation and living conditions (85, 86). From this perspective, the health status, psychological well-being, and confidence in the future associated with sports participation may be more closely related to fertility intentions among unmarried young adults.

Third, the heterogeneity observed by hukou status and educational attainment suggests that the association between sports participation and fertility intentions may be embedded within broader social-resource and health-behavior environments. Previous studies have documented differences across urban–rural and hukou groups in access to public sports resources, opportunities for sports participation, and levels of physical activity in China. Rural areas generally have more limited sports facilities and professional exercise guidance, whereas urban hukou status has been associated with higher levels of physical activity participation (87, 88). Higher educational attainment has also been associated with greater health awareness, stronger capacity to acquire exercise-related knowledge, and more regular sports participation (89). In this context, sports participation among individuals with higher educational attainment and relatively greater social resources may more often reflect an orientation toward proactive health management and long-term quality of life (90), providing one possible explanation for the hukou- and education-related heterogeneity observed in this study. Nevertheless, these interpretations remain potential explanations rather than established mechanisms and should be further examined by incorporating factors such as access to public sports resources, employment characteristics, income stability, and family support.

### 4.6 Theoretical contributions

First, this study broadens the explanatory scope of research on fertility intentions among young adults. Previous studies have primarily examined fertility intentions from the perspective of structural factors such as income, housing, employment stability, marital status, and family policies. By incorporating sports participation as a health-promoting behavior into the analytical framework, the present study identified a stable but modest positive association between sports participation and fertility intentions. This finding suggests that understanding fertility intentions among young adults requires attention not only to external resources and institutional constraints but also to health-related behaviors and overall life well-being.

Second, this study extends the discussion of the public health relevance of sports participation. Research on sports and public health has traditionally focused on physical health, chronic disease prevention, mental health, social interaction, and quality of life. The present study further considers sports participation within the broader contexts of family formation and population health. The findings suggest that the health-related, psychological, and social resources associated with sports participation may also be relevant to how young adults evaluate their future family lives. Accordingly, the public health significance of sports participation may extend beyond disease prevention and health-related outcomes to include broader dimensions of well-being and long-term life planning among young adults.

Third, this study provides further evidence regarding the links among sports participation, fertility intentions, and positive psychological resources. Life satisfaction and confidence in the future jointly accounted for 30.61% of the total association, suggesting that young adults’ evaluations of their current lives and expectations regarding future development represent important psychological dimensions for understanding the association between sports participation and fertility intentions. In particular, the serial pathway from “current life evaluation” to “future development expectations” suggests that fertility planning among young adults may be related to both current life experiences and longer-term expectations about the future. This finding deepens the understanding of fertility intentions from the perspectives of psychological well-being and health promotion and provides new empirical evidence linking health-related behaviors, psychological resources, and family development.

Finally, this study further highlights the public health relevance of low fertility. Low fertility concerns not only population size and age structure but is also closely related to physical health, mental health, social well-being, family quality of life, and reproductive health among young adults. Sports participation should therefore not be regarded as an independent instrument for promoting fertility. Rather, it may be more appropriately positioned as one component of a broader system for supporting youth health and well-being, while further attention should be given to the potential complementarities among health-promotion policies, reproductive health services, and family-support policies.

### 4.7 Practical implications

The policy relevance of this study does not lie in treating sports participation as a direct instrument for increasing fertility. Rather, the findings suggest that physical health, psychological well-being, quality of life, and a sense of future security among young adults should be considered important components of a fertility-friendly social environment. Fertility intentions are associated with multiple economic, familial, sociocultural, and institutional factors. Accordingly, sports-promotion measures should not be viewed as substitutes for childcare support, housing security, employment stability, or work–family balance policies, but rather as one component of a broader system supporting the health and well-being of young adults.

First, greater coordination between sports promotion and youth health and reproductive health services should be considered. Alongside continued improvements in community-based “15-minute fitness circles,” public sports facilities, and affordable exercise services, evidence-based physical activity guidance could be appropriately incorporated into premarital health services, preconception health education, youth health management, and primary public health services. Collaboration among community health institutions, public sports organizations, and youth service agencies may help extend sports services beyond the provision of exercise facilities toward more integrated support for physical, mental, and reproductive health. Second, greater attention should be given to the psychological well-being of young adults, as reflected in life satisfaction. Universities, communities, healthcare institutions, and employers could consider incorporating regular physical activity into mental health and stress-management programs. Exercise guidance, group-based sports, and community activities may provide accessible opportunities for stress regulation, social interaction, and health support. Such measures should also be integrated with psychological counseling, social support, employment services, and family support, so that complex fertility-related pressures are not reduced to individual differences in physical activity or psychological status. Third, greater attention should be paid to young adults’ expectations regarding long-term life stability. The mediating role of confidence in the future suggests that fertility support for young adults should not rely solely on short-term health-promotion measures, but should also consider social conditions related to employment, housing, childcare, caregiving responsibilities, and work–family balance that are closely associated with perceived future security. Sports and health-promotion policies may therefore be more appropriately coordinated with employment protection, housing support, affordable childcare, reproductive health services, and family-friendly policies to provide broader support for young adults’ current quality of life and expectations about the future. Fourth, the differentiated needs of different groups of young adults should be considered. Given that the association between sports participation and fertility intentions varied by sex, hukou status, educational attainment, and marital status, youth health-promotion policies may benefit from avoiding a fully homogeneous service model. Greater attention could be given to reproductive health, career development, and caregiving pressures among young women; healthy lifestyles, social connectedness, and future expectations among unmarried young adults; and equitable access to sports and health services among groups with relatively limited resources.

### 4.8 Limitations and future perspectives

Several limitations should be acknowledged. First, although this study used panel data from the 2018 and 2022 waves of the China Family Panel Studies (CFPS), together with two-way fixed-effects models, lagged sports participation, and multiple robustness checks, its observational design does not permit strict causal inference. Fixed-effects models can account for time-invariant individual heterogeneity but cannot fully eliminate time-varying unobserved confounding, such as changes in family values, economic expectations, psychological states, or major life events. Likewise, lagged analyses cannot completely resolve potential endogeneity. The findings should therefore be interpreted as relatively robust longitudinal statistical associations. Future studies could use additional survey waves and stronger identification strategies, such as natural experiments or instrumental-variable approaches. Second, sports participation was measured differently in the 2018 and 2022 CFPS waves. To improve comparability, the measure was harmonized as a binary indicator, which inevitably resulted in the loss of information on frequency, intensity, and duration. The baseline findings should therefore not be interpreted as dose–response relationships. Although the 2022 exercise-duration sensitivity analysis produced broadly consistent results, future research should employ standardized measures that capture physical activity frequency, intensity, duration, and type. Third, sports participation, life satisfaction, confidence in the future, and fertility intentions were mainly self-reported, and some variables were measured using single items, making them potentially susceptible to recall bias, social desirability bias, and measurement error. In addition, the present study examined only two psychological pathways. Although life satisfaction and confidence in the future jointly accounted for 30.61% of the total association, other mechanisms may also be relevant. Future studies could incorporate multidimensional psychological measures, objective physical activity indicators, and additional factors such as physical health, resilience, self-efficacy, social support, relationship quality, and work–family conflict. Fourth, the study was conducted within China’s specific sociocultural and policy context. Cross-national differences in family welfare, employment protection, childcare provision, gender roles, and reproductive health policies may be associated with different patterns linking sports participation, psychological well-being, and fertility intentions. Cross-national and cross-institutional comparative studies are therefore needed to assess the cross-cultural stability of the observed associations. Finally, because only two survey waves were available, the serial mediation findings reflect statistical relationships consistent with the proposed theoretical ordering but do not establish the temporal sequence of sports participation, life satisfaction, confidence in the future, and fertility intentions. Future research using three or more waves and longitudinal approaches such as cross-lagged or random-intercept cross-lagged panel models could provide stronger evidence regarding directionality.

Despite the limitations noted above, this study has several strengths. First, the nationally representative CFPS panel data provide broad population coverage, offering a robust empirical basis for examining the association between sports participation and fertility intentions among young adults across diverse regional, hukou, and socioeconomic backgrounds. Second, the individual fixed-effects approach accounts for time-invariant individual heterogeneity, thereby enhancing the robustness of the estimates. In addition, the analyses of the independent and serial mediating pathways involving life satisfaction and confidence in the future provide further evidence regarding the potential psychological relevance of current life evaluations and future expectations in this association. The heterogeneity analyses further suggest that the association may vary across sociodemographic groups. Overall, this study extends the understanding of fertility intentions among young adults from the perspectives of health-related behavior and psychological well-being and supports considering sports participation within a broader framework of youth health, psychological well-being, and family development. These findings also provide an empirical basis for future research using longer-term longitudinal designs, cross-cultural comparisons, and stronger causal-identification strategies.

## 5 Conclusion

Using panel data from the 2018 and 2022 waves of the China Family Panel Studies (CFPS), this study systematically examined the association between sports participation and fertility intentions among young adults, as well as the potential psychological pathways underlying this association. The main findings were as follows: (1) sports participation was significantly and positively associated with fertility intentions among young adults; (2) life satisfaction and confidence in the future independently mediated the association between sports participation and fertility intentions; and (3) life satisfaction and confidence in the future also formed a significant serial mediating pathway between sports participation and fertility intentions.

Overall, the primary contribution of this study lies in linking a modifiable health-promoting behavior with fertility intentions among young adults and in further identifying a serial psychological pathway involving life satisfaction and confidence in the future. In doing so, the study extends the public health framework for understanding fertility intentions from the perspective of psychological well-being. It should be emphasized, however, that the findings represent statistical associations rather than strict causal relationships. Sports participation should therefore not be regarded as an independent instrument for promoting fertility, but may be more appropriately positioned as one component of a broader youth health-promotion framework, coordinated with mental health services, reproductive health services, employment and housing support, affordable childcare, and work–family balance policies. Future research could use longitudinal data from additional waves, objective measures of physical activity, and stronger causal-identification designs to further examine the directionality of the relationships among sports participation, psychological resources, and fertility intentions, as well as the mechanisms underlying these associations.

## Data Availability

Publicly available datasets were analyzed in this study. This data can be found at: https://www.isss.pku.edu.cn/cfps/.
