# Supplementary material for: The association between sports participation and fertility intentions among Chinese youth: an empirical study based on the China family panel studies
Source: Front Public Health. 2026 Oct 2;14:1904111. doi: 10.3389/fpubh.2026.1904111 (PMC13672502; doi:10.3389/fpubh.2026.1904111)
Supplement: Supplementary file 1 [file Data_Sheet_1.PDF]

Supplementary Material

1 Supplementary Figures and Tables

1.1 Supplementary Figures

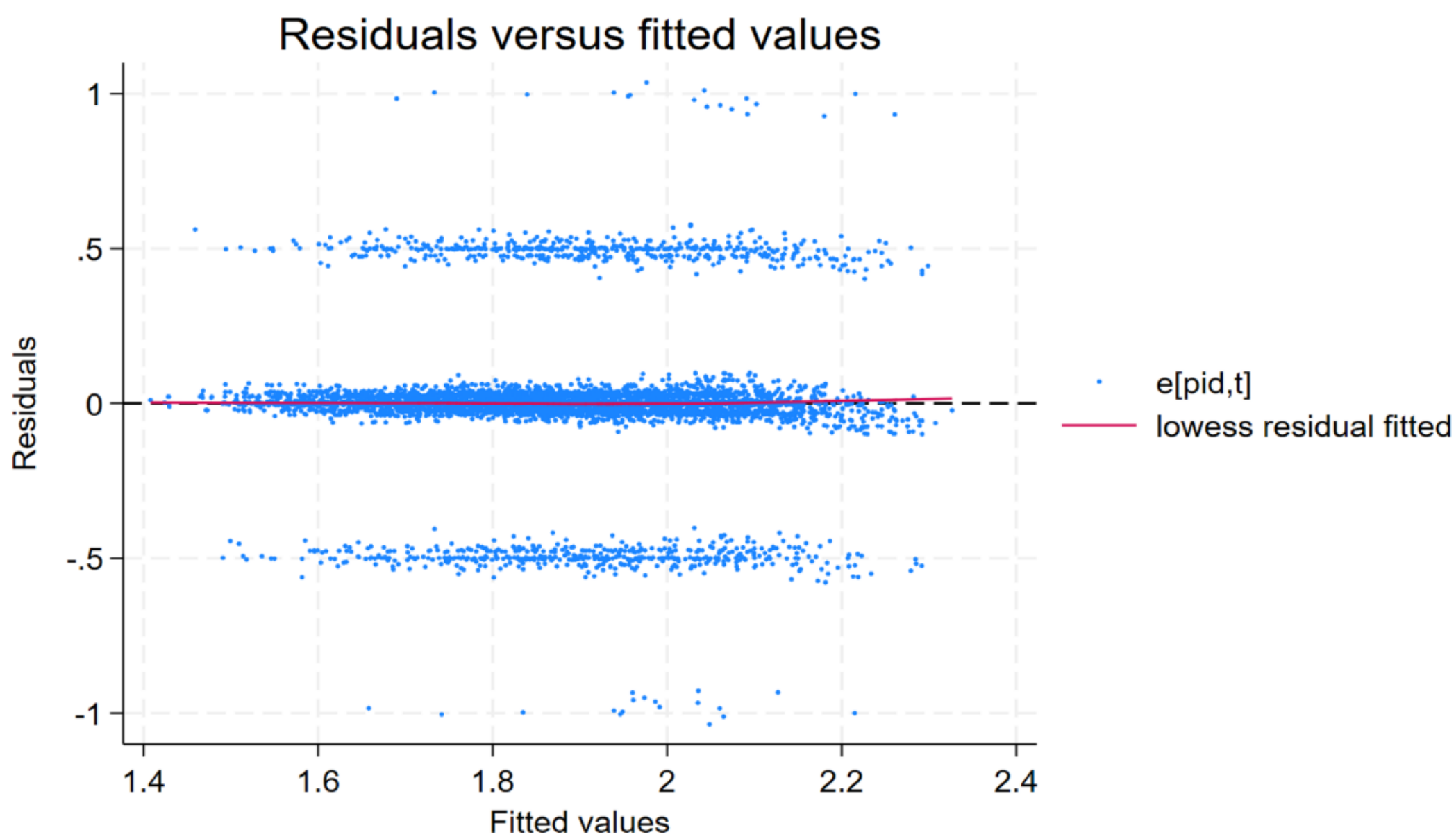

Supplementary Figure S1: Residuals versus fitted values with LOWESS smoothing.

The residual-versus-fitted plot shows that the LOWESS smoothed curve remained close to the zero reference line across most of the fitted-value range, with no pronounced systematic curvilinear pattern. This provides no clear evidence of substantial nonlinearity in the model specification. The horizontal banding of the residuals is consistent with the discrete nature of the fertility-intention measure.

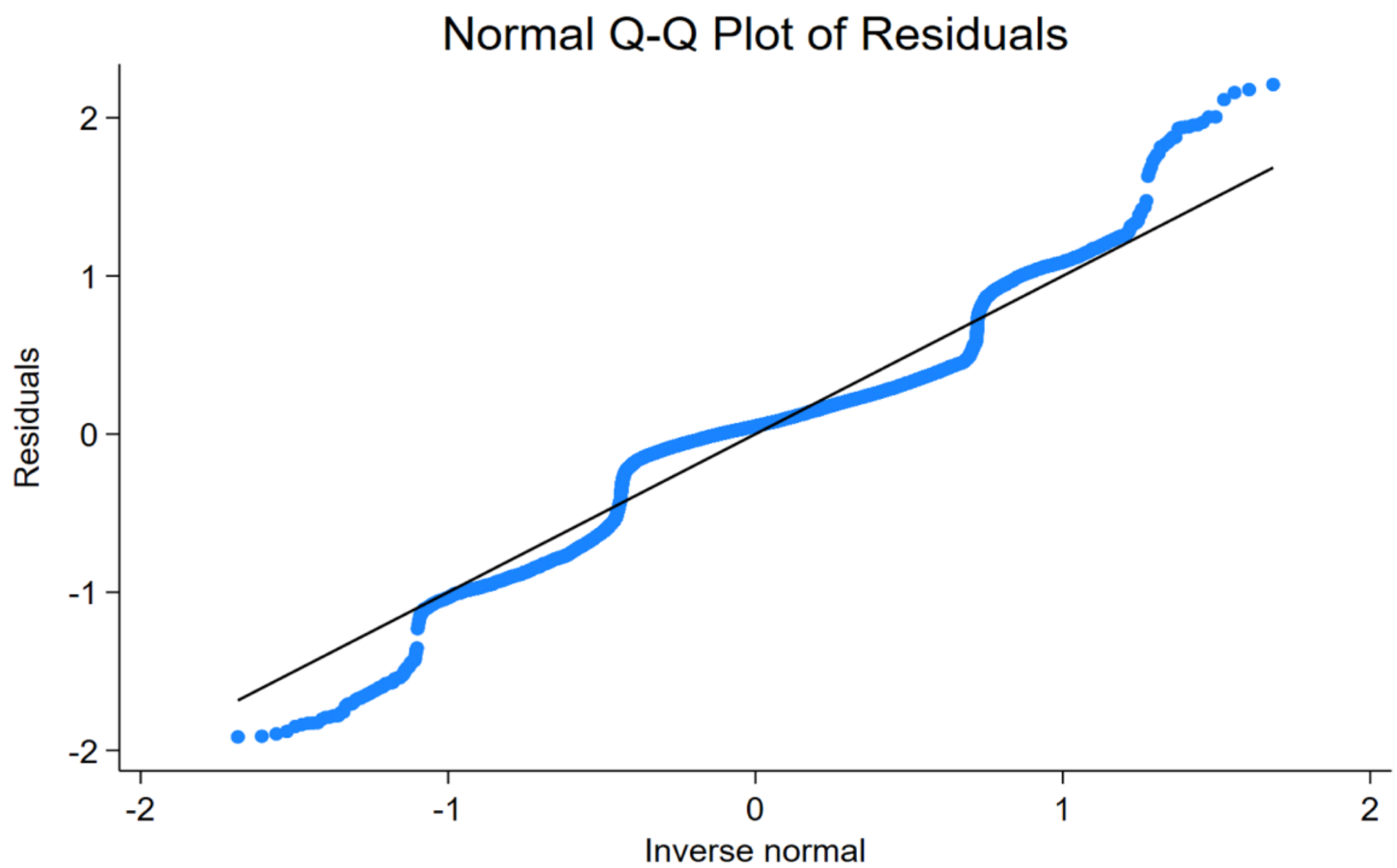

Supplementary Figure S2. Normal Q–Q plot of the regression residuals.

The normal Q–Q plot shows a stepwise pattern and noticeable deviations of the residuals from the reference line, particularly in the tails, indicating departures from normality. This pattern is partly consistent with the discrete nature of the fertility-intention measure. The graphical evidence is consistent with the results of the joint skewness–kurtosis test reported in Supplementary Table S2.

1.2 Supplementary Tables

The variance inflation factor (VIF) values for the variables included in the model ranged from 1.08 to 1.77, with a mean VIF of 1.36. All VIF values were well below the commonly used threshold of 5, indicating no evidence of serious multicollinearity among the explanatory variables.

Supplementary Table S1 Multicollinearity diagnostics based on variance inflation factors

**Variables**	VIF
Life satisfaction	1.77
Confidence in the future	1.73
Education	1.50
Age	1.46
Marital status	1.38
Hukou status	1.27
Subjective social status	1.15
Sports participation	1.15
Self-rated health	1.13
income	1.08
Mean VIF	1.36

The joint skewness–kurtosis test was statistically significant, $\chi^2(2) = 421.53$, $p < 0.001$, indicating that the regression residuals deviated from normality. In addition, the Breusch–Pagan/Cook–Weisberg test was statistically significant, $\chi^2(1) = 484.71$, $p < 0.001$, rejecting the null hypothesis of constant variance and indicating the presence of heteroskedasticity.

Supplementary Table S2：Model diagnostic tests

Diagnostic test	Statistic	p-value	Interpretation
Joint skewness–kurtosis test	$\chi^2(2) = 421.53$	$p<0.001$	Residuals deviate from normality
Breusch–Pagan/Cook–Weisberg test	$\chi^2(1) = 484.71$	$p<0.001$	Heteroskedasticity is present

Overall, the model diagnostic results showed no evidence of serious multicollinearity or substantial systematic nonlinearity. However, the residuals deviated from normality, and the formal

heteroskedasticity test indicated non-constant error variance. Given the large sample size and the panel structure of the data, individual-level cluster-robust standard errors were used for statistical inference to account for heteroskedasticity and within-individual dependence arising from repeated observations. Taken together, these diagnostic results support the use of the specified linear model with cluster-robust inference, while the departures from normality and heteroskedasticity were explicitly accounted for in the estimation strategy.
